# Biomechanical evaluation of the human mandible after temporomandibular joint replacement under different biting conditions

**DOI:** 10.1038/s41598-021-93564-3

**Published:** 2021-07-07

**Authors:** Manuel Pinheiro, Robin Willaert, Afaq Khan, Anouar Krairi, Wim Van Paepegem

**Affiliations:** 1 Department of Materials, Textiles and Chemical Engineering, Mechanics of Materials and Structures (MMS), Ghent University, Ghent, Belgium; 2 Department of Oral and Maxillofacial Surgery, University Hospitals Leuven, Leuven, Belgium; 3 Department of Head and Neck Surgery, Ghent University Hospital, Ghent, Belgium; 4 Materials Innovation Institute (M2i), Delft, The Netherlands; 5 Department of Biomechanical Engineering, Delft University of Technology, Delft, The Netherlands

**Keywords:** Medical research, Engineering

## Abstract

Temporomandibular joint (TMJ) replacement with an implant is only used when all other conservative treatments fail. Despite the promising short-term results, the long-term implications of TMJ replacement in masticatory function are not fully understood. Previous human and animal studies have shown that perturbations to the normal masticatory function can lead to morphological and functional changes in the craniomaxillofacial system. A clearer understanding of the biomechanical implications of TMJ replacement in masticatory function may help identify design shortcomings that hinder their long-term success. In this study, patient-specific finite element models of the intact and implanted mandible were developed and simulated under four different biting tasks. In addition, the impact of re-attaching of the lateral pterygoid was also evaluated. The biomechanics of both models was compared regarding both mandibular displacements and principal strain patterns. The results show an excessive mediolateral and anteroposterior displacement of the TMJ implant compared to the intact joint in three biting tasks, namely incisor (INC), left moral (LML), and right molar (RML) biting. The main differences in principal strain distributions were found across the entire mandible, most notably from the symphysis to the ramus of the implanted side. Furthermore, the re-attachment of the lateral pterygoid seems to increase joint anteroposterior displacement in both INC, LML and RML biting while reducing it during LGF. Accordingly, any new TMJ implant design must consider stabilising both mediolateral and anteroposterior movement of the condyle during biting activities and promoting a more natural load transmission along the entire mandible.

## Introduction

The temporomandibular joint (TMJ) can be affected by multiple disorders, which can cause pain, decreased joint movement and functional disability during chewing, swallowing and speech. The replacement of the TMJ with an alloplastic joint can be recommended when all other conservative treatments fail, particularly in patients with structural joint disorders and a history of pain and dysfunction^1^.

Three main TMJ replacement systems are currently available, namely the patient-fitted TMJ Concepts system (Ventura, CA, USA), the stock/custom Biomet Microfixation systems (Jacksonville, FL, USA), and the stock/patient-specific Nexus CMF systems (Salt Lake City, UT, USA). Recent meta-analyses showed similar outcomes regarding a decrease in pain scores and improvements in function, diet, and maximum incisal opening for both stock and patient-specific implants^2,3^. Short-term follow-up studies on TMJ replacement systems have shown a survival rate ranging from 67 to 97% after 3-years^4,5^. However, mid to long-term results are yet unclear. For instance, Biomet Microfixation systems have shown mixed mid-term results, with good functional results after 8-years^6^ and a failure rate of 11% after 7-years follow-up^7^. The main complications were heterotopic bone formation, infection, hardware loosening or failure, and increased pain scores^7,8^. However, long-term studies are not available. The only long-term study reported was conducted for the TMJ Concepts system and had a follow-up period of 21 years. The study showed encouraging results regarding survival, material wear and functional outcomes^9^. However, these results were obtained for a small group of 56 patients, which corresponded to only 50% of the initial cohort. Thus, all existing designs seem to provide sub-optimum outcomes, and the need for more independent research on the long-term performance of the different TMJ prostheses was recently emphasized^8^.

The mandible experiences different biomechanical stimuli during jaw opening and biting^10^. Biomechanical and morphological studies found a strong correlation between muscle cross-sectional area (which is approximately proportional to muscle force generation) and mandibular morphology, especially concerning the shape of the ramus and coronoid process^11^. In addition, animal studies showed that a decrease in functional demands could lead to structural changes in both muscles and mandibular bone, with bone loss being observed in underloaded regions^12,13^. Thus, the preservation of appropriate masticatory function seems crucial for maintaining mandibular bone health and mass. This observation may be critical in patients with temporomandibular disorders (TMD). Muscle recruitment in TMD patients remains controversial, with some studies claiming no significant differences^14,15^, while other studies showing substantial differences^16,17^ between pathological cases and controls. In either case, the use of an incorrectly designed alloplastic joint replacement may further compromise mandibular biomechanics and hinder the long-term success of TMJ implants.

Different aspects of the human mandible biomechanics have been studied: (1) the *in-vivo* kinematics of the TMJ replacement during mouth opening, protrusion and closing^18–21^; (2) the biomechanics of the intact mandible and TMJ disk during biting^22–24^; (3) TMJ implant design and safety^25,26^. However, the biomechanical effect of TMJ replacement during different biting conditions is yet to be studied. In this study, finite element (FE) modelling was used to understand the biomechanical behaviour of the implanted mandible and compare it with the intact mandible, concerning both joint movements and principal strain distributions. The TMJ design used here was inspired in a commercially available design and patient-fitted to the target anatomy. Identifying design limitations is a fundamental step towards the development of better TMJ implants, which can improve their long-term outcomes.

## Methods

**Figure 1:**
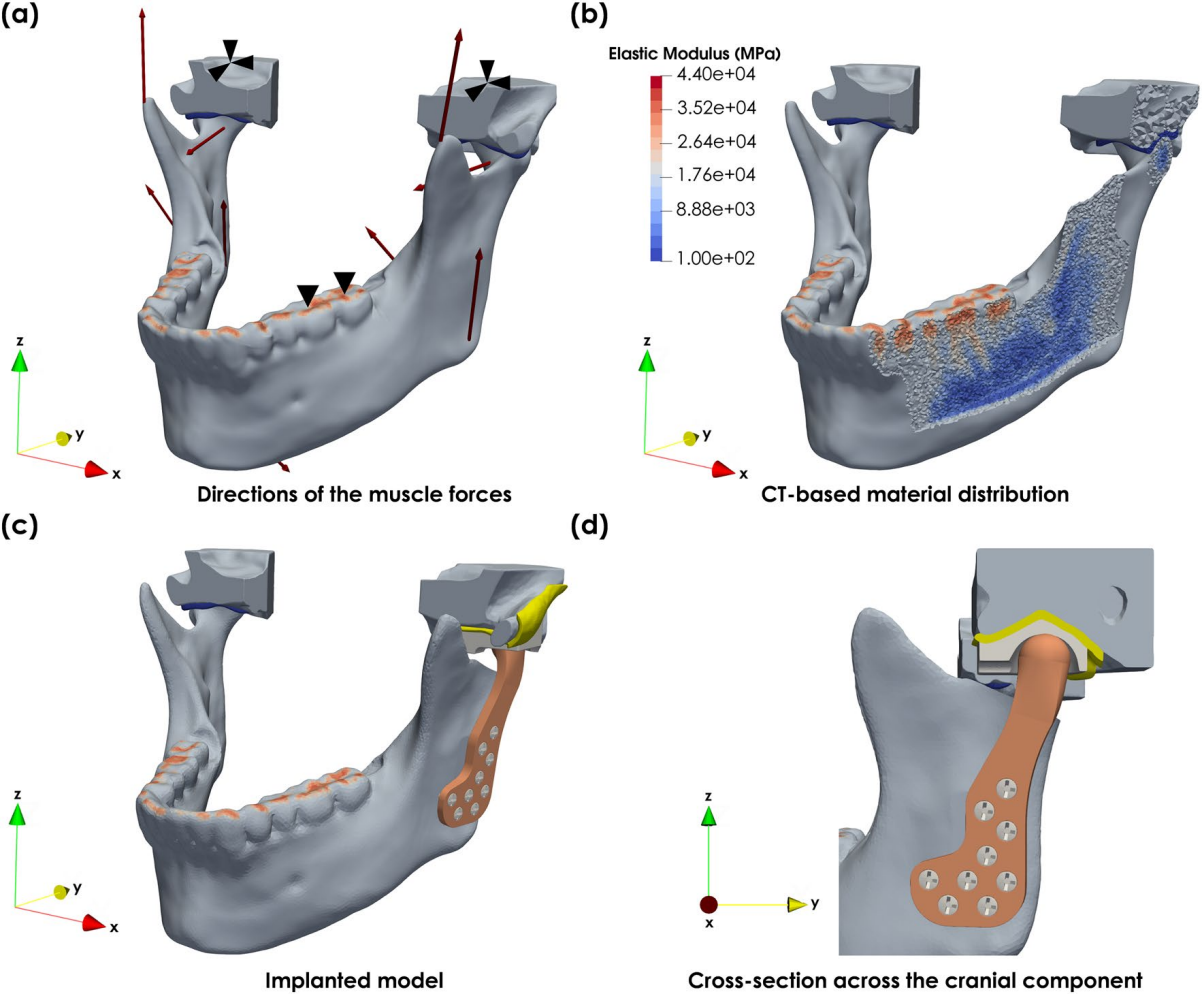
Patient-specific model of the human mandible: (**a**) musculoskeletal model of the intact mandible, (**b**) greyscale-based material distribution across the intact mandible, (**c**) finite element model of the mandible with the patient-specific implant and (**d**) cross-section along the mid-plane of the scustomised cranial component of TMJ replacement system.

A randomly selected and anonymised computed tomographic (CT) data with a spatial resolution of 0.52 × 0.52x1.0 mm was obtained for this study. Data collection was approved by the institutional review board (B670201733474), and the participant provided written informed consent allowing it to be used for research purposes. Segmentation of the intact mandible was performed in Materialise Mimics v21.0 (Materialise Inc., Leuven, Belgium) and refined as proposed in^27^ (Fig. 1a, b). Finite element (FE) mesh generation was conducted in Materialise 3-Matic v14.0 (Materialise Inc., Leuven, Belgium), whereas FE analysis and post-processing were performed with Abaqus CAE 2019 (Abaqus Inc., USA) and Paraview 5.8.0 (Kitware, 2020).

### Implant design and finite element meshing

Improper alignment of the TMJ implant may have detrimental implications in the magnitude and direction of the joint reaction force and implant stability and wear of the fossa component^5^. The 3D model was positioned in the natural head position as proposed in^28^ to ensure an appropriate alignment between the implant with the target anatomy.

The design of the patient-specific implant was performed in SolidWorks (Dassault Systems, 2018). The shape of the implant was inspired by the Biomet Microfixation systems design. Implant customisation entailed the definition of a proximal coronal curvature to approximate the original centre of rotation of the TMJ, adjustment of the implant length to preserve the original muscular lever arms and positioning of the fixation screws away from the mandibular nerve (Fig. 1c). The cranial fossa component was designed as a purely spherical joint^29^, with clearance between the articulating surfaces (Fig. 1d).

FE meshing of the intact and implanted models was performed considering quadratic tetrahedral elements (C3D10). For the intact model, convergence in the nodal solutions was achieved with a surface maximum edge length of 0.50 mm, corresponding to 3,446,040 nodes and 2,448,785 elements. The same FE meshing parameters were used to obtain the FE mesh of the implanted model.

### Model boundary conditions

The intact and implanted mandibles were simulated considering four biting tasks, namely incisal (INC), left group (LGF), left molar (LML) and right molar (RML) biting^22^. The bite forces documented in the literature for human subjects range from 200 to 1500 N^30^. While, the maximum bite forces for INC, LGF, RML and LML are 570 N^31^, 1336 N^32^ and 911 N^31^, respectively. However, to simulate the mandible under more common loading conditions, a set of nominal forces was derived from the maximum values. The nominal bite forces were defined as 50% of the maximum values, i.e., 285 N for INC, 668 N for LGF and 455.5 N for LML and RML.

The muscle activations were obtained from the musculoskeletal model proposed by Korioth et al. (1994) and uniformly scaled until the desired bite force was measured at the dental arch. Table 1 lists the muscle force components to produce the desired nominal bite forces for each biting task.

**Table 1:** Muscle forces for nominal incisal biting (INC), right molar (RMOL) and left group biting (LGF).

Muscle name	INC			RMOL			LGF		
	Fx (N)	Fy (N)	Fz (N)	Fx (N)	Fy (N)	Fz (N)	Fx (N)	Fy (N)	Fz (N)
Right masseter	− 60.91	− 54.175	185.785	− 48.305	− 29.105	132.435	− 49.595	− 31.135	137.32
Left masseter	60.91	− 54.175	185.785	40.255	− 24.255	110.36	51.62	− 8.59	117.305
Right temporalis	− 8.435	10.91	41.705	− 32.345	51.655	150.175	− 8.625	13.975	39.905
Left temporalis	8.435	10.91	41.705	27.18	42.53	126.375	86.765	147.54	393.855
Right lat. pterygoid	90.965	− 100.695	− 16.045	10.105	− 12.14	− 2.79	17.065	− 19.125	− 3.255
Left lat. pterygoid	− 90.965	− 100.695	− 16.045	− 21.89	− 26.305	− 6.045	− 65.24	− 74.945	− 14.38
Right med. pterygoid	147.525	− 113.225	240.11	57	− 43.75	92.775	144.035	− 110.545	234.425
Left med. pterygoid	− 147.525	− 113.225	240.11	− 40.715	− 31.25	66.27	− 13.265	− 10.18	21.59
Right ant. digastric	− 10.875	41.89	− 10.56	–	–	–	− 8.28	31.9	− 8.045
Left ant. digastric	10.875	41.89	− 10.56	–	–	–	11.115	42.815	− 10.795

The FE model was constrained at whereas the superior surfaces of the two cranial components were constrained in all directions, whereas the biting points were constrained in the Oz direction (Fig. 1a). In addition, frictionless contact was assigned to both condyle-cartilage and mandible-implant interfaces, while the implant-liner interaction was modelled as frictional $\left( {\mu = 0.05} \right)$.

In the implanted model, the interactions between the TMJ disk and mandibular fossa, the screws and the mandible, the screws and implant, the liner and fossa component were modelled as tied constraints (Fig. 1c, d). The left masseter muscle was considered fully functional^33^, and two variations of the musculoskeletal model were tested. In the first, the left lateral pterygoid muscle was removed, while in the second, the lateral pterygoid was re-attached as proposed in^34^. To simulate an imperfect connection between tissue and implant, the force generated by the muscle was limited to 50% of the healthy condition.

### Assignment of material properties

The mandible was modelled as isotropic, non-homogeneous and linear elastic material, where the material properties were derived from the CT image data. The non-homogeneous material assignment was performed in Materialise Mimics (Materialise Inc., Leuven, Belgium). All other materials were set as homogeneous, isotropic and linear elastic.

Due to the inexistence of specific material mapping laws for the mandible and the lack of appropriate CT calibration data, the non-homogeneous material assignment was performed considering theoretical values for the density $\left( {\rho ^{T} } \right)$ and elastic modulus $\left( {E^{T} } \right)$ for each biological material. Different power laws were computed for trabecular bone $\left( {E_{t}^{P} } \right)$, cortical bone $\left( {E_{c}^{P} } \right)$, dentin $\left( {E_{d}^{P} } \right)~$ and enamel $\left( {E_{e}^{P} } \right)$. Each law was adjusted to produce a range of *in-vivo* elastic moduli in agreement with literature values. The range of elastic properties was assigned as follows. The elastic moduli for trabecular bone $\left( {E_{t}^{P} } \right)$ followed the power law:

1 $$E_{t}^{P} = 0.00040{\rho _{t}^{P}}^{2.01}$$

where the material density $\left( {\rho _{t}^{P} } \right)$ was set to be within the range of $\left[ {0,{\text{~}}1000} \right]\;{\text{kgm}}^{{ - 3}}$. Equation 1 was applied to the voxels with greyscale values from $\left[ { - 1000,\;~500} \right]~{\text{HU}}$. The final range of elastic moduli was $E_{t}^{P} = \left[ {191,~374} \right]~{\text{MPa}}$, which is within the theoretical values for trabecular bone (Table 2). Similarly, for cortical bone $\left( {E_{c}^{P} } \right)$ the elastic moduli were defined according to:

2 $$E_{c}^{P} = 0.0050{\rho _{c}^{P}}^{2.01}$$

**Table 2:** Mechanical properties for the different tissue types considered in the finite element simulations.

Tissue	Theoretical modulus ($E^{T}$ (MPa))	Theoretical density ($\rho ^{T}$ (${\text{kgm}}^{{ - 3}}$))	CT density ($\rho ^{P}$ (${\text{kgm}}^{{ - 3}}$))	CT values (HU)	Elastic moduli laws	Practical modulus ($E_{{\left\{ {t,c,d,e} \right\}}}^{P} ~$ (MPa))	Poisson’s ratio (ν)	References
Trabecular bone	180–380	$50~ \le \rho < 1000$	$0~ \le \rho < 1000$	− 1000–500	$E_{T} = 0.00040{\rho _{t}^{P}}^{2.01}$	191–374	0.300	^51^
Cortical bone	11,300–22,900	$1779 \le \rho < ~~2000$	$1001 \le \rho < ~~2000$	501–1500	$E_{C} = 0.0050{\rho _{c}^{P}}^{2.01}$	7320–18,140	0.300	^22,51^
Dentin	10,200–29,000	$2480 \le \rho < ~~2900$	$2001 \le \rho < ~~2480$	1501–2000	$E_{D} = 0.0045{\rho _{d}^{P}}^{2.01}$	21,030–28,040	0.300	^51,52^
Enamel	20,000–91,100	$2500 \le \rho < ~~2924$	$2481 \le \rho < ~~2924$	2001–4000	$E_{E} = 0.0050{\rho _{e}^{P}}^{2.01}$	35,230–43,980	0.300	^51,52^
Ti-6Al-4 V	113,800	4420	–	–	–	–	0.342	^53^
Co-Cr	210,000	10,000	–	–	–	–	0.290	^51,52^
UHMWPE	1258	940	–	–	–	–	0.460	^54^
TMJ disk	15.8–65.0	–	–	–	–	45.0	0.400	^55,56^

where the material density $\left( {\rho _{c}^{P} } \right)$ was set to be within [1001, 2000] ${\text{kgm}}^{{ - 3}}$ and the corresponding greyscale values were $\left[ {501,~1500} \right]~{\text{HU}}$.

The range of CT values for dentin was set to be $\left[ {1501,~2000} \right]~{\text{HU}}$ and the elastic moduli were defined as:

3 $$E_{d}^{P} = 0.0045{\rho _{d}^{P}}^{2.01}$$

whereas for the enamel, the CT values range from $\left[ {2001,~4000} \right]~{\text{HU}}$ and the elastic moduli were given by:

4 $$E_{e}^{P} = 0.0050{\rho _{e}^{P}}^{2.01}$$

The range of theoretical densities and elastic moduli and the final range of elastic moduli for each material are summarised in Table 2. The final material distribution is displayed in Fig. 1b.

### Computation of principal strain patterns

The principal strain distributions denoted as $\varepsilon _{{dist}}$ along the intact and implanted mandibles were computed for each load case according to:

5 $$\varepsilon _{{dist}} = \left\{ {\begin{array}{*{20}l} {\varepsilon _{I} ,} \hfill & {if~\left| {\varepsilon _{I} } \right| \ge \left| {\varepsilon _{{III}} } \right|} \hfill \\ {\varepsilon _{{III}} ,} \hfill & {if~\left| {\varepsilon _{I} } \right| < \left| {\varepsilon _{{III}} } \right|} \hfill \\ \end{array} } \right.$$

where $\varepsilon _{I}$ is the first principal strain and $\varepsilon _{{III}}$ the third principal strain $\left( {\varepsilon _{{III}} \le \varepsilon _{{II}} \le \varepsilon _{I} } \right)$. $\varepsilon _{{dist}}$ indicates what regions are mainly under tension or compression. For comparison purposes, the principal strain distributions in the intact mandible $\left( {\varepsilon _{{dist}}^{{int}} } \right)$ were mapped onto the nodes of the implanted mandible $\left( {\hat{\varepsilon }_{{dist}}^{{int}} } \right)$. The differences between principal strain distributions were computed according to:

6 $$\varepsilon _{{dist}}^{{dif}} = sign\left( {\hat{\varepsilon }_{{dist}}^{{int}} } \right)sign\left( {\varepsilon _{{dist}}^{{imp}} } \right)\left| {\hat{\varepsilon }_{{dist}}^{{int}} - \varepsilon _{{dist}}^{{imp}} } \right|$$

The value of $\varepsilon _{{dist}}^{{dif}}$ is proportional to $\left| {\hat{\varepsilon }_{{dist}}^{{int}} - \varepsilon _{{dist}}^{{imp}} } \right|$ and is positive when both intact and implanted mandibles are under tension or compression and negative when maximum principal strains have opposite signs.

## Results

### Displacements and deformations of the intact and implanted mandibles

**Figure 2:**
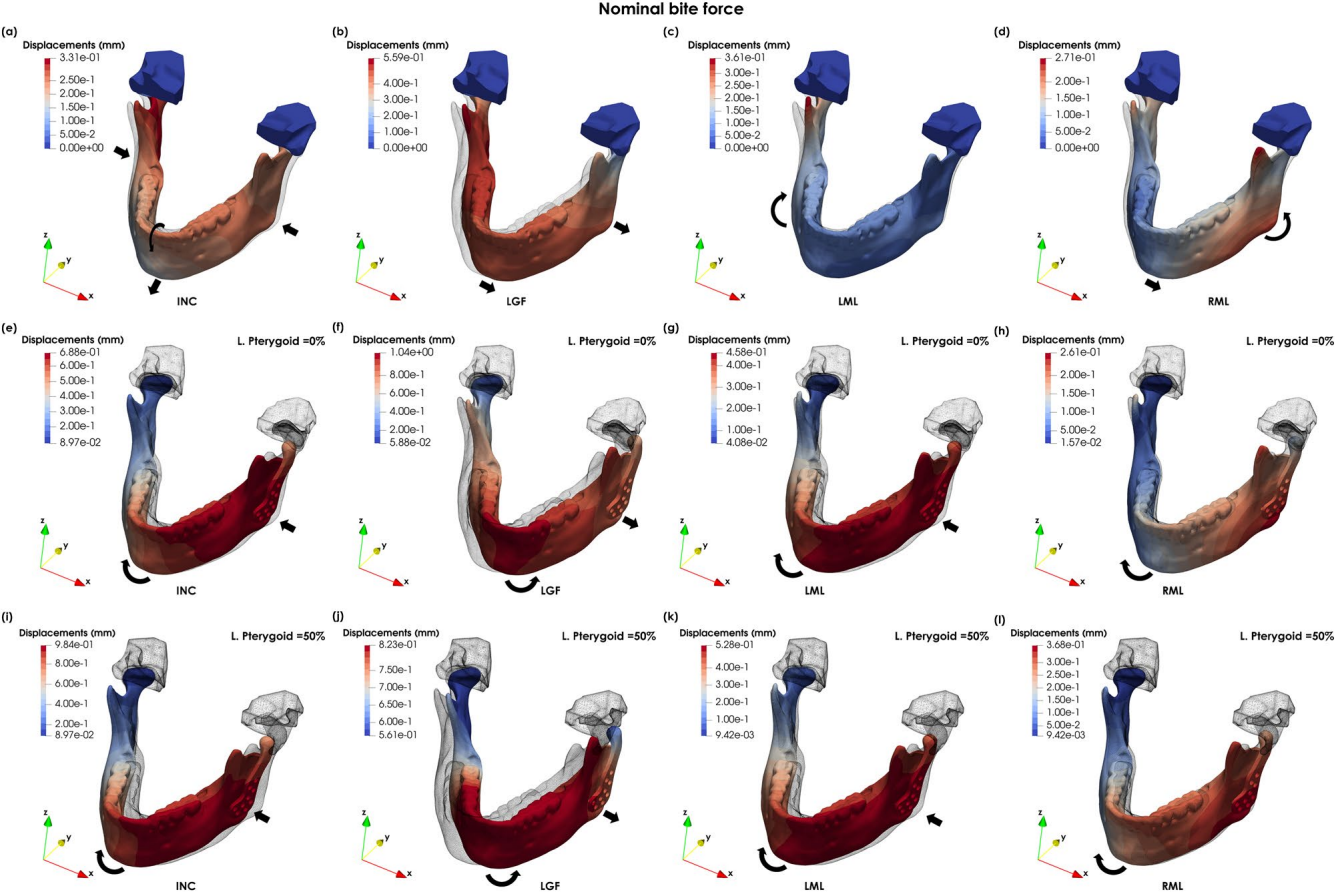
Magnitude of the displacements in the intact mandible during (**a**) INC (scaling factor × 15), (**b**) LGF (scaling factor × 15), (**c**) LML (scaling factor × 20) and (**d**) RML (scaling factor × 20); magnitude of the displacements in the implanted mandible during (**e**) INC (scaling factor × 15), (**f**) LGF (scaling factor × 10), (**g**) LML (scaling factor × 15), (**h**) and RML (scaling factor × 15) for the implanted mandible; (**i**) INC plus 50% lateral pterygoid (scaling factor × 15), (**j**) LGF plus 50% lateral pterygoid (scaling factor × 10), (**k**) LML plus 50% lateral pterygoid (scaling factor × 15), (**l**) and RML plus 50% lateral pterygoid (scaling factor × 15) for the implanted mandible (equivalently deformed intact mandible displayed as wireframe).

Figure 2a–d shows the displacements of the intact mandible for incisor (INC), left group (LGF), left molar (LML) and right molar (RML) bite forces. Whereas Fig. 2e–l show the displacements of the implanted mandible without and with lateral pterygoid re-attachment, respectively, against the intact mandible for all load cases.

**Figure 3:**
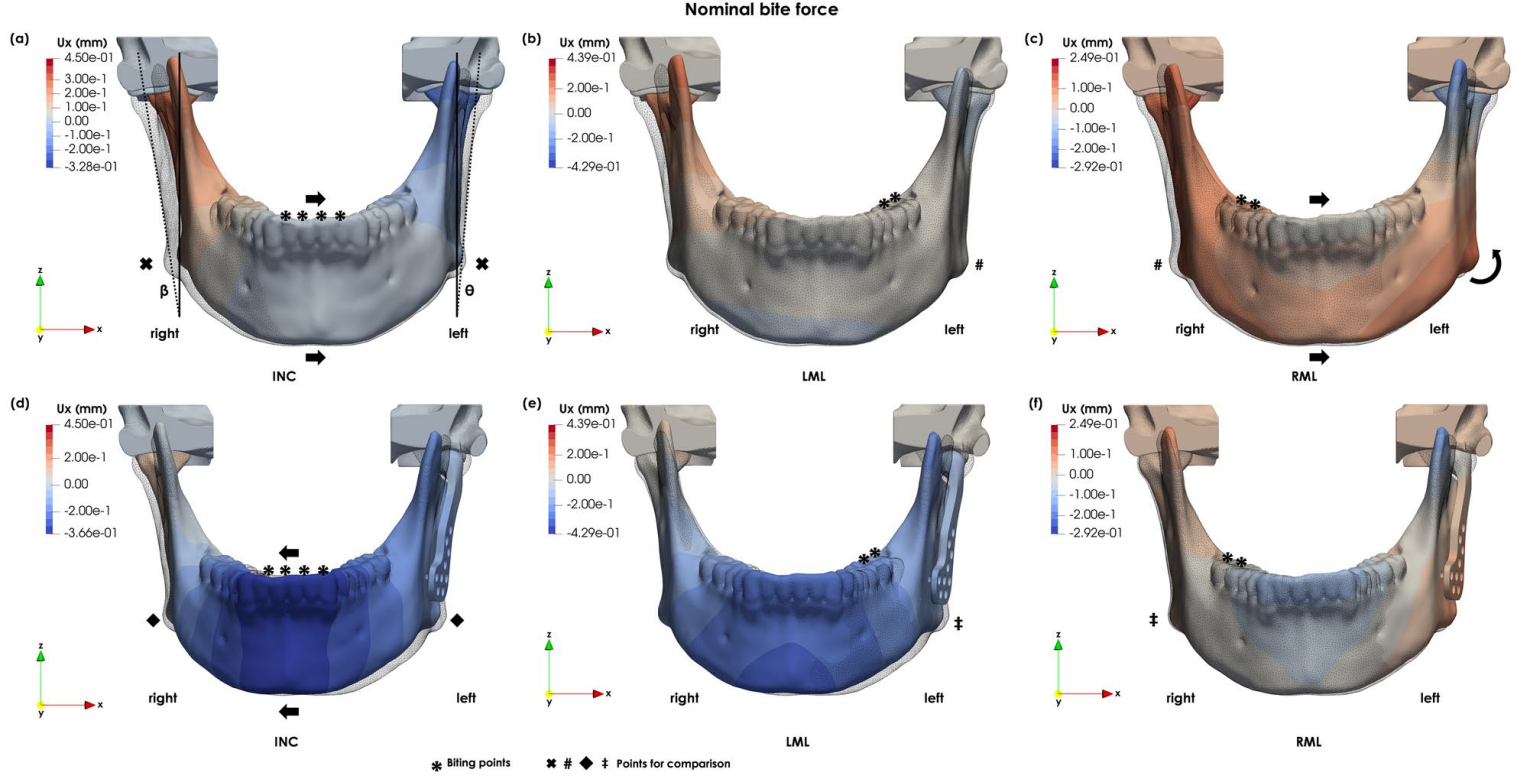
Medio-lateral displacements of the intact mandible for (**a**) INC, (**b**) LML al and (**c**) RML, and for the implanted mandible (**d**) INC, (**e**) LML and (**f**) RML (scaling factor × 20).

There is an almost symmetrical displacement forward and compression of the gonial area during INC loading (Fig. 2a). In LFG, the mandible moves towards the working (left) side (Fig. 2b), whereas during LML and RML there is a slight displacement forward, combined with a rotation towards the working side (Fig. 2c, d). There is a tendency for the mandible to deviate towards the left side. This can be observed not only from the mediolateral (Ux) displacement during INC (Fig. 3a) but also by comparing the mediolateral displacements during LML and RML (Fig. 3b, c).

In the implanted mandible, the implanted side moves forward and rotates towards the healthy side during INC loading (Figs. 2e and 3d). In LFG, there is a displacement towards the left side combined with a posterior rotation of the condyle (Fig. 2f). Whereas in LML, there is a forward displacement of the implanted side and a rotation towards the healthy side (Figs. 2g and 3e). During RML, the mandible shows a slight movement forward and a small rotation towards the right side (Figs. 2h and 3f). The displacement for the intact and implanted mandibles are very similar during RML.

The re-attachment of the lateral pterygoid muscle reduces the displacements of the mandible during LGF (Fig. 2j), however it increases the mandibular displacements in the implanted condyle for all the other biting tasks. (Fig. 2i, k–l).

Dental arch deformations also change from the intact to the implanted case. Table 3 summarises the dental arch deformations for the four biting tasks. In INC and LGF biting, there is an increase of dental arch/mandibular mediolateral contraction from the canines to the gonial area. In LML the maximum dental arch contraction was observed around the second molars. Whereas in RML, there is a contraction if the dental arch combined with dilation of the gonial area. The deformations of the implanted mandible are lower by several orders of magnitude when compared with the deformations of the intact model. For instance, during INC the canines move towards the midline (compression) by 16.7 µm, whereas after TMJ replacement, they dilate by approximately 0.10 µm.

**Table 3:** Dental arch deformations for INC, LGF, LML and RMOL for the intact mandible under nominal bite forces (positive values of deformation imply compression).

Biting task	Deformation intact mandible (µm)				Deformation implanted mandible (µm)			
	Canines	2nd Premolars	2nd Molars	Gonial area	Canines	2nd Premolars	2nd Molars	Gonial area
INC	16.7	58.3	145.1	269.1	− 9.930e−02	1.102e−01	− 2.479e−01	− 5.474e−02
LGF	7.6	24.4	60.1	66.1	− 5.4	3.567e−01	− 3.1	1.3
LML	7.8	27.4	56.1	12.4	1.039e−02	− 6.637e−03	− 5.800e−02	− 4.333e−02
RML	8.8	25.1	49.7	− 11.9	− 7.085e−02	4.583e−02	− 1.866e−01	− 1.890e−01

### Principal strain distribution across intact and implanted mandible

**Figure 4:**
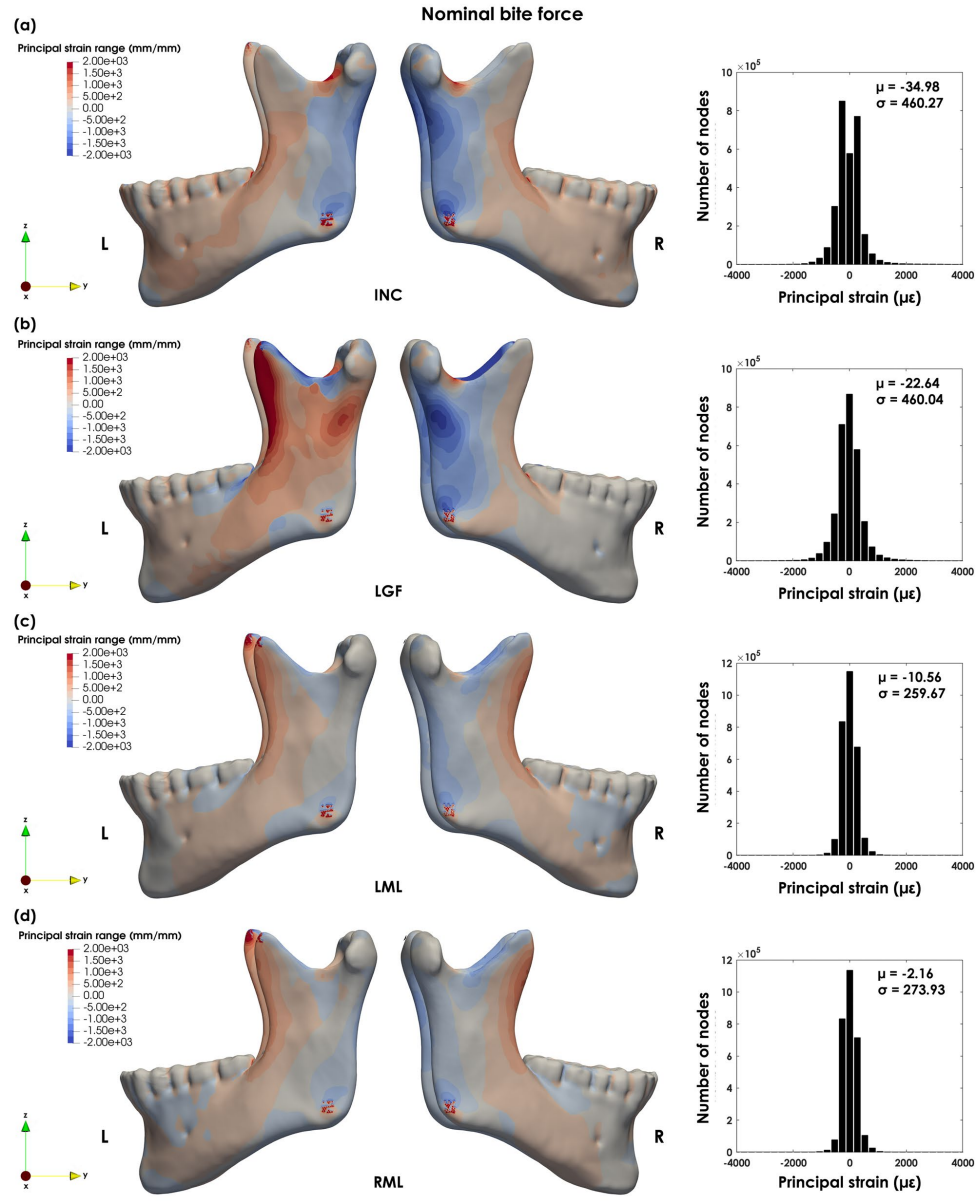
Lateral view of the principal strain distribution during (**a**) incisor biting (INC), (**b**) left group biting (LGF), (**c**) left molar biting (LML) and (**d**) right molar biting (RML) for the intact mandible.

**Figure 5:**
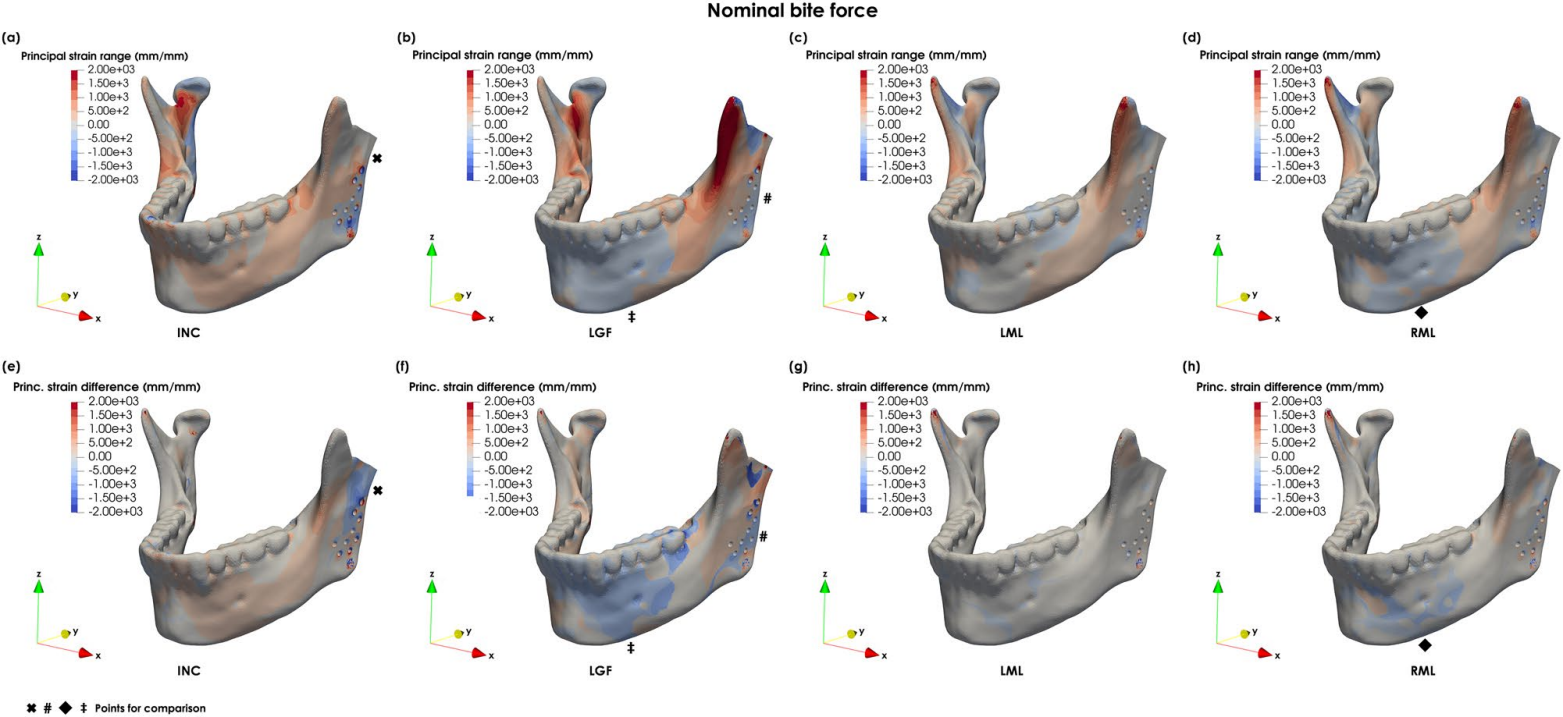
Principal strain distribution during (**a**) incisor biting (INC), (**b**) left group biting (LGF), (**c**) left molar biting (LML) and (**d**) right molar biting (RML) for the implanted mandible and principal strain differences $\varepsilon _{{dist}}^{{dif}}$ between intact and implanted mandible under (**e**) incisor biting (INC), (**f**) left group biting (LGF), (**g**) left molar biting (LML), and (**h**) right molar biting (RML) (with not lateral pterygoid activation).

The principal strain distributions $\left( {\varepsilon _{{dist}} } \right)$ across the intact mandible are presented in Fig. 4a–d. The principal strain patterns of the implanted mandible and principal strain differences are shown in Fig. 5a–h.

In INC, the maximum compressive principal strains were found in the posterior surface of the rami $\left( { - 1700\mu \varepsilon } \right)$. The maximum tensile strains were observed at the mandibular notch $\left( {2500\mu \varepsilon } \right)$ and at the oblique line $\left( {900\mu \varepsilon } \right)$. Principal strain values in the remainder of the mandible range from $\left[ { - 800,~800} \right]\mu \varepsilon$ (Fig. 4a). High compressive strains were also observed at the posterior lower lingual surface of the mandibular body and rami and the chin. Tensile strains were also found at the buccal surface of the mandibular body and lingual and lower surface of the symphysis.

During LGF, the principal tensile strains were observed at the left oblique line, lateral surface of the left ramus and medial surface of the right mandibular notch and oblique line. The maximum tensile strains $\left( {3250\mu \varepsilon } \right)$ were found in the left temporal muscle insertion. The maximum compressive strains $\left( { - 1850\mu \varepsilon } \right)$ were observed at the lateral surface of the right ramus, left mandibular notch and medial surface of the coronoid process (Fig. 4b).

The maximum principal strains values during LML and RML were lower when compared to INC and LGF. The principal strain values were mostly within the $\left[ { - 500,~500} \right]\mu \varepsilon$ range (Fig. 4c, d). In LML, the lateral surface of the body and oblique line and medial surface of the notch were under tension. On the contrary, the medial surface of the body and ramus and posterior surfaces of the coronoid processes were under compression (Fig. 4c). The maximum tensile strains $\left( {1200~\mu \varepsilon } \right)$ were found at the anterior surface of the left ramus, whereas the maximum $\varepsilon _{{III}}$ were observed at the posterior border of the right ramus $\left( { - 790\mu \varepsilon } \right)$ and coronoid processes $\left( { - 990\mu \varepsilon } \right)$. Similarly to LML, in RML, the maximum $\varepsilon _{I}$ were observed along the anterior surface of the rami $\left( {1260\mu \varepsilon } \right)$, while the maximum $\varepsilon _{{III}}$ occurred along the posterior surface of the right coronoid processes $\left( { - 1240\mu \varepsilon } \right)$ and left ramus $\left( { - 750\mu \varepsilon } \right)$ (Fig. 4d). In both molar cases, compressive strains were observed immediately below the molars on the working side, whereas tensile strains were observed on the balancing side.

Both principal strain plots and principal strain differences $\left( {\varepsilon _{{dist}}^{{dif}} } \right)$ show the changes in loading between the intact and implanted case and that these propagate across the entire mandible (Fig. 5a–h). The main differences between the intact and implanted mandible were observed for INC, LGF and RML. In INC, the main differences were observed in the implanted ramus around the screws, especially around the most proximal and distal screws (Fig. 5a, e). In INC and RML $\varepsilon _{{dist}}^{{dif}}$ were observed almost exclusively on the implanted side from the symphysis to the ramus. Whereas in LGF, they were found across the entire mandible (although higher on the implanted side). In the proximal ramus, the negative differences below the resected condyle show that there is an inversion of the biomechanical stimulus from compressive to tensile (Fig. 5e). Furthermore, in LGF there is a compressive rather than tensile stimulus in the lower surface of the implanted ramus and anterior mandibular body (Fig. 5b, f).

Minimal principal strain differences were observed in LML (Fig. 5c, g), while in RML the region around the left mental foramen changed from tension in the intact model to compression in the implanted mandible (Fig. 5d, h).

## Discussion

In TMD management, the replacement of the TMJ with an implant is only recommended in patients with structural joint disorders and a history of pain and dysfunction and when all other conservative treatments fail^1^. Different biomechanical aspects of the human mandible have been studied before, such as TMJ implant design and safety^25,26^, the kinematics of the intact and implanted bone^18–21^, and the behaviour of the intact mandible and the TMJ disk under different loading conditions^22–24^. However, the biomechanical effects of TMJ replacement during different biting conditions were not yet addressed. Hence, given the importance of proper masticatory function in the maintenance of mandibular bone health and volume^12,13^, a clearer understanding of the main biomechanical differences between intact and implanted mandible may help to identify the limitations of current TMJ replacement systems. Also, FE models can help to predict the impact of different surgical techniques in mandibular biomechanics, such as the re-attachment of the lateral pterygoid.

Four biting tasks were considered, namely INC, LGF, LML and RML. For the intact mandible, the displacements patterns are consistent with Korioth et al. (1994) observations for all loading conditions. In Korioth et al. (1994), the maximum mandibular displacements are $0.620\;{\text{mm}}$ in middle incisors in INC, $0.920\;{\text{mm}}$ at the right central incisor in LGF, and $1.060\;{\text{mm}}$ at the left gonial angle in RML. Maximum displacements in this report are smaller despite the higher bite forces. This may be explained by the morphological differences between the two models since mandibular movement is highly dependent on bone density and volume^10^.

The mandible tends to move towards the left side. The FE simulations show larger mediolateral displacements of the right ramus than the left side (Fig. 3a). Likewise, in RML the mandible moves mediolaterally towards the balancing side^22^ (Fig. 3c), however the reverse is not observed during LML (Fig. 3b). This observation could be induced by the morphological asymmetry of the mandible. The mandibular symphysis deviates to the left side, the left ramus is oriented more vertically, and the left body is thicker and more pronounced anteriorly than the right side (Fig. 3a). These morphological findings are related to a unilateral dental cross-bite^35,36^. The results suggest that mandibular asymmetry influences the displacement patterns during mastication: the mandible moves towards the cross-bite side, independent of the chewing side.

The translational movements are often the most affected after TMJ replacement, especially during maximum jaw opening^21^. The main factors contributing to the reduction of mandibular translation are thought to be the detachment of the lateral pterygoid muscle and the geometry of the articular surface^19,21,37^ and articular and muscular tissue fibrosis^18,38^. After surgery, the recovery of some degree of joint translation has been attributed to compensatory mechanisms, such as the recruitment of the suprahyoid, masseter, and medial pterygoid muscles^18^, to gravity and the increase in motion on the healthy joint (the so-called mandible *pseudo-translation*)^39^. Mandible *pseudo-translation* was also attributed to the slightly inferior placement of the centre of rotation of the implanted joint when compared to the healthy joint^19^. Furthermore, a kinematic study with unilateral TMJ prosthesis showed that maximum mouth opening was obtained with a significant lateral deviation towards the implanted side^20^. These observations may be associated with the stabilising role of the lateral pterygoid, which prevents the condyle from moving laterally and ultimately leading to the translation of the mandible towards the implanted side.

To the best of our knowledge, no *in-vivo* studies were conducted to assess the movement of the implanted TMJ during biting and assess the isolated role of the lateral pterygoid muscle in condyle stabilisation. The role of the lateral pterygoid muscle is still controversial^40,41^. In EMG studies, the lateral pterygoid muscle is often described as having an active role in both jaw opening and closing movements, where the superior head is active during jaw closing, and the inferior head is active during opening. Theoretically, during mastication, the lateral pterygoid helps to move the mandible inward and forward inside the cranial fossa, placing the condyle in a locked position while controlling the horizontal movement of the jaw^42^. In the implanted joint, the mandible can move more freely both in the mediolateral and anterior–posterior directions. Contrarily to jaw opening, the FE simulations during INC, LML and RML chewing show a forward movement and a rotation of the mandible towards the healthy side. During LGF, there is a lateral displacement and posterior rotation of the implanted condyle, which could be explained by the absence of the lateral pterygoid and the high forces generated by the left temporal and right medial pterygoid muscles.

In the musculoskeletal model^22^, the combined action of the superior and inferior heads of the lateral pterygoid move the condyle forward, inward and downward, effectively constraining the lateral movement of the condyle and unloading the temporomandibular disk during biting. The re-attachment of the lateral pterygoid seems to be effective during LGF, however the same was not observed for INC, LML and RML. Often TMJ replacement designs allow the forward translation of the joint to facilitate mouth opening^26^. Here, the results obtained indicate that even with a spherical joint, excessive forward movement is observed during INC, RML and LML and stress the importance of designing a fossa component that acts as a mechanical constraint to the forward movement of the mandible during biting. Recently, promising results were obtained after the re-attachment of the lateral pterygoid in sheep^43^. Post-operative complications resumed to instability between the fossa component and the supporting bone but not at the alloplastic joint^43^. These seemingly contradictory results may be explained by the morphological differences between humans and sheep. A morphological assessment of the TMJ demonstrated that the articular tubercle is rudimentary in sheep because condylar movement is mainly mediolateral. On the contrary, humans have more prominent articular tubercles, and the articular movement is mostly anteroposterior^44^. These differences in morphology may explain why the results obtained by the FE model predict an increase in joint instability after lateral pterygoid re-attachment.

Concerning the response of the bone during biting, several studies have evaluated the deformations of the mandible *in-vivo*, showing that the symphysis remains stable and considerable deformations occur at the body and ramus. The mandible exhibits a significant deformation towards the midline during mouth opening and protrusion, with deformation amplitudes up to 800 µm at the first molars and up to 1500 µm at the rami^10^. Similarly, during unilateral and bilateral biting, dental arch deformations ranging from $10~\left( { \pm \,14} \right)$ to $340~\left( { \pm \,28} \right)$ µm were observed in the premolar region^21^. Korioth et al. (1994) demonstrated that mandibular deformations at the dental arch increased anteroposteriorly, with the maximum values ranging from 100 µm for RML and LGF to 200 µm for INC in the second molar area^22^. Here, a similar behaviour was observed for the intact mandible. However for the implanted mandible, the deformations are very small and possibly negligible.

Meyer et al. (2002) used photo-elasticity to analyse the surface strains in human mandibles during RML. The observations were consistent: (a) compressive strain patterns in the posterior and tensile patterns in the anterior surfaces of the rami; (b) compressive strains continued anteriorly along the lower border of the body, whereas tensile strains continued anteriorly along the upper border of the body; (c) tensile strains were observed below the mandibular notch; and (d) compressive and tensile strains were found along the posterior and anterior surfaces of the condylar neck, respectively^45^. Gröning et al. (2013) found that during incisor, canine and molar biting, the highest principal strains were found below the molars, oblique line, base of the mandibular body, and posterior margin of the ramus below the condyles. The remaining areas of the mandibular ramus showed low principal strains^46^. Here, the FE predictions are in agreement with previous observations for all four load cases. In LGF, the posterior aspect of the left ramus is under tension, possibly due to the extremely high muscle forces generated by the ipsilateral temporalis, which drive the deformation of the mandible towards the working/active side.

Normal peak physiological strains in adult load-bearing bones can range from $2500\mu \varepsilon$ in tension to $4000\mu \varepsilon$ in compression. In human mandibles loaded with an artificial bite force of 600 N the principal strains up to $800\mu \varepsilon$ were observed along the corpus^47^. Likewise, in FE analysis of the mandible during premolar and molar biting (552.6 N), the surface strain distribution along mid-corpus ranged from approximately $100 - ~750\mu \varepsilon$, whereas the maximum surface strains $\left( {1250\mu \varepsilon } \right)$ were observed in the alveolar area^48^. Gröning et al. (2013) reported principal strain values higher than $\pm 1500\mu \varepsilon$ under different load cases. The authors stressed that the values observed may be underestimated since no extreme bite loads were considered^46^. Finally, FE analysis combined with *in-vivo* surface strains measured showed that the critical threshold for induced bone resorption in the mandible might be around $3600\mu \varepsilon$ ^49^.

The principal strains in this study were within the expected values. The exception was at the insertion of the left temporal muscle in LGF biting, where a maximum tensile strain of $3250\mu \varepsilon$ was observed. The simplification of the insertion area may explain this observation since the full force generated by the muscle is concentrated in a small area. Nevertheless, the maximum values did not exceed the $3600\mu \varepsilon$ proposed in^49^. Very high principal strains were also observed along the lateral edge of the in the first proximal screw during INC and LGF (about $- 8400\mu \varepsilon$ and $9530\mu \varepsilon$). Similarly to the muscle insertion areas, these values may be due to the simplification of the bone-screw interface. However, more accurate predictions of the true $\varepsilon _{I}$ and $\varepsilon _{{III}}$ would imply the explicit modelling of the screw hole and thread.

Alterations in bone volume across the mandible were previously observed in rabbits treatment with botulinum toxins to inactivate the masseter muscle. A considerable amount of bone loss was observed at the condylar process of the injected side and molar regions on both sides of the mandible^13^. Here, the primary principal strain differences between intact and implanted models were observed along the proximal lateral, distal lateral and posterior surfaces of the implanted ramus and the buccal surface of the mandibular body. Furthermore, an inversion of the nature of the strain stimulus was observed at the posterior surface of the ramus (INC) and between the symphysis and the first molar region (LGF and RML).

In the current study, a FE model of the intact mandible was developed and validated against *in-vivo, *in vitro* and post mortem* data available in the literature. TMJ replacement was simulated, and the results obtained in the implanted mandible were compared with the intact one to identify the main differences regarding mandibular displacements and principal strains. However, the model possesses several limitations. The use of a post-calibration procedure of the CT data allows for modelling of the different tissues but not in a fully patient-specific manner. However, the results show that this may be a reliable option when no CT calibration is available. The musculoskeletal model was based on the work of Korioth et al. (1994) and adjusted to the current anatomy. Given the limited data on individual muscle function in TMD patients, no muscle unbalance was modelled between the healthy and affected side. In Korioth et al. (1994), the combined action of the two muscle heads leads to a downward pull of the condyle by the lateral pterygoid. Therefore, the results obtained after lateral pterygoid re-attachment are limited by the current musculoskeletal model.

Nevertheless, the results show that lateral pterygoid re-attachment may lead to joint instability after TMJ replacement. The re-attachment of the lateral pterygoid may be possibly done on a patient-to-patient basis, especially with the widespread in-house custom-made implant production^50^. Finally, a spherical condylar component^29^ can restrict condylar movement compared to other designs^25,26^ where a certain amount of translational movement is allowed. Therefore, a careful analysis of the kinematic behaviour of the glenoid fossa component should be considered in the design phase.

In conclusion, the results indicate that during INC, LML and RML biting, the implanted mandible tends to move towards the healthy side, whereas during LGF it moves towards the implanted side. In addition, there is an excessive joint movement both mediolaterally and anteroposteriorly during biting. These observations agree with the theoretical role of the lateral pterygoid, which controls the fine horizontal movements of the jaw. The results obtained show that the re-attachment of this muscle after TMJ surgery, as recently proposed in a clinical report^34^ may be important for controlling the posterior movement during LGF. However, it seems to have little effect on the stabilisation of the forward movement in the remaining load tasks (INC, RML and LML). These observations emphasise the need to re-design the articular surface of the cranial component in future designs. Furthermore, the principal strain plots show that the main differences occurred along the proximal ramus and were more pronounced during INC, LGF and RML. New TMJ designs should provide more natural joint movement and load transmission between the implant and the supporting bone. The evaluation of new TMJ designs may also imply the development of an appropriate framework for quantitative biomechanical performance comparison between concurrent solutions.
